# Editorial for the Specific Issue of Radioprobes and Other Bioconjugates for Cancer Theranostics

**DOI:** 10.3390/cancers16030541

**Published:** 2024-01-26

**Authors:** Yasushi Arano

**Affiliations:** Chiba University, Chiba 263-8522, Japan; arano@chiba-u.jp

Theranostics refers to the systematic integration of targeted diagnostics and therapeutics, which promotes precise and personalized cancer treatment. A variety of theranostic pairs have been proposed and evaluated so far, including SPECT/PET imaging and targeted radionuclide therapy, and fluorescent/photoacoustic imaging and photodynamic/chemodynamic/thermodynamic therapy. Diagnostic imaging before treatment allows for the selection of patients who would benefit from the treatment. Diagnostic imaging post therapy enables the efficacy of the treatment to be evaluated. This concept is not new, as exemplified by the treatment of thyroid cancer with [^131^I]NaI, a b and g emitter [1]. ^111^In- and ^90^Y-labeled monoclonal antibodies against CD20 (Zevalin) are another example of radiotheranostic pairs. ^111^In emits g rays for diagnosis, while ^90^Y emits a b ray for radiotherapy, and the two metallic radionuclides are incorporated into the monoclonal antibody against CD 20 conjugated with a benzyl-DTPA derivative for complexation [2]. Numerous efforts have been made to develop new theranostic pairs, including radiolabeled unsymmetric urea derivatives for prostate cancer [3] and synthetic somatostatin analogs for neuroendocrine tumors [4]. The development of new theranostic pairs involves the integration of multidisciplinary approaches, including new targeting molecules, drug delivery systems, radiochemistry, and conjugation chemistry between parental targeting devices and payloads (e.g., radionuclides (radiometal chelates), fluorescent dyes, and cytotoxic drugs). Four original manuscripts in this Special Issue present important findings that will be useful for the future development of theranostic pairs.

A variety of fluorescent dyes have been developed and used to label biomolecules, such as proteins and peptides, for in vitro characterization, in vivo molecular imaging, and future applications in theranostics. Fluorescent dyes are usually highly lipophilic due to their large p-conjugated systems. They also have charged groups to improve their stability in aqueous solutions. When the dyes are conjugated to a biomolecule of interest, the overall lipophilicity and net charge of the conjugates are altered. However, little is known about the effect of dye conjugation on the PK/PD of the resulting conjugates. Takakura et al. used cyanine dyes with different chemical properties and investigated the tumor cell uptake of a cell-penetrating peptide (CPP) in vitro and in vivo after conjugation with the dyes. They observed that the chemical properties of fluorescent dyes played a significant role in the in vitro tumor cell binding and in vivo accumulation in mouse tumors [5].

Several hydrophilic cationic amphiphilic peptides (CAPs) exhibit antitumor activity since the membrane surface of tumor cells tends to possess negative charge. Fuchigami et al. chose SVS-1 (KVKVKVKVpPTKVKVKVK-NH_2_: KV6) as a lead peptide, where two (KV)_3_ sequences are connected via a b-turn tetrapeptide (VDpPT). They synthesized KV derivatives with shorter (KV4) and longer (KV8) KV sequences. They also synthesized cationic peptides by substituting lysine in KV6 with arginine (RV6) or histidine (HV6). The zeta potential, circular dichroism, and cell binding of the peptides were compared. In vivo tumor accumulation of the peptides was also assessed in the murine tumor model after NOTA conjugation and subsequent radiolabeling with ^67^Ga. The KV length and lysine substitution exhibited significant differences in tumor accumulation [6].

Sano et al. proposed a combination of a near-infrared (NIR) light irradiation-sensitive drug carrier and a thermosensitive polymer as a new tumor-specific drug delivery system. Gold nanorods (GNRs) are gold nanoparticles that exhibit a moderate temperature rise upon NIR light irradiation with excellent photothermal conversion efficiency. Polyoxazoline (POZ) is a thermosensitive drug carrier with a low critical solution temperature. Their design involved (1) the delivery of GNRs to tumors, (2) irradiation with NIR light to increase the intratumor temperature, and (3) the administration of POZ so that the polymer would efficiently be taken up by the tumor via polymer aggregation. To prove the concept of this design, the GNR was intratumorally injected. Then, ^111^In-labeled POZ was intravenously injected into mice pre-irradiated with or without NIR light [7].

Astatin-211 (^211^At) is obtained from neutral bismuth targets via ^209^Bi(a, 2n)^211^At in a cyclotron, which renders the radiohalogens attractive radionuclides for targeted alpha therapy. Hanaoka et al. previously developed 2-[^211^At] astato-a-methyl-phenylalanine (AAMP) and found that AAMP was incorporated into tumor cells via L-type amino acid transporter 1 (LAT1), which is highly expressed in various types of human tumors. While AAMP was stable against in vivo dehalogenation, its therapeutic effect was insufficient due to low tumor retention. The authors speculated that the tumor retention of AAMP would be prolonged by inhibiting the urinary excretion of the compound. The authors evaluated the effect of preloading with probenecid, an inhibitor of organic anion transporters, on the tumor retention of AAMP in a mouse model [8].

I sincerely hope that the manuscripts in this Special Issue will inspire readers in their research. 
